# Advances in Delivery Mechanisms of CRISPR Gene-Editing Reagents in Plants

**DOI:** 10.3389/fgeed.2022.830178

**Published:** 2022-01-24

**Authors:** Larissa C. Laforest, Satya Swathi Nadakuduti

**Affiliations:** ^1^ Plant Molecular and Cellular Biology Program, University of Florida, Gainesville, FL, United States; ^2^ Department of Environmental Horticulture, University of Florida, Gainesville, FL, United States

**Keywords:** gene-editing, CRISPR-Cas9, gene targeting, agrobacterium-mediated transformation, biolistics, protoplasts, nanoparticles

## Abstract

Gene-editing by CRISPR/Cas systems has revolutionized plant biology by serving as a functional genomics tool. It has tremendously advanced plant breeding and crop improvement by accelerating the development of improved cultivars, creating genetic variability, and aiding in domestication of wild and orphan crops. Gene-editing is a rapidly evolving field. Several advancements include development of different Cas effectors with increased target range, efficacy, and enhanced capacity for precise DNA modifications with base editing and prime editing. The existing toolbox of various CRISPR reagents facilitate gene knockouts, targeted gene insertions, precise base substitutions, and multiplexing. However, the major challenge in plant genome-editing remains the efficient delivery of these reagents into plant cells. Plants have larger and more complex genome structures compared to other living systems due to the common occurrence of polyploidy and other genome re-arrangements. Further, rigid cell walls surrounding plant cells deter the entry of any foreign biomolecules. Unfortunately, genetic transformation to deliver gene-editing reagents has been established only in a limited number of plant species. Recently, there has been significant progress in CRISPR reagents delivery in plants. This review focuses on exploring these delivery mechanisms categorized into *Agrobacterium*-mediated delivery and breakthroughs, particle bombardment-based delivery of biomolecules and recent improvements, and protoplasts, a versatile system for gene-editing and regeneration in plants. The ultimate goal in plant gene-editing is to establish highly efficient and genotype-independent reagent delivery mechanisms for editing multiple targets simultaneously and achieve DNA-free gene-edited plants at scale.

## Introduction

CRISPR/Cas9 derived from *Streptococcus pyogenes* (SpCas9) is the most used gene-editing reagent in plants. Unlike its predecessors, zinc finger nucleases (Gao et al., 2010; Osakabe et al., 2010; Zhang et al., 2010) and Transcription Activator-like Effector Nucleases (TALENs) (Cermak et al., 2011; Li et al., 2012), which rely on protein-based DNA recognition mechanisms, CRISPR/Cas systems are RNA-guided endonucleases. The resulting versatility, simplicity, and cost-effectiveness brought about by CRISPR led to significant advances in plant genome engineering. In the CRISPR/Cas9 system, a chimeric single guide RNA (sgRNA), formed by fusion of CRISPR RNA (crRNA) and a *trans*-activating crRNA (tracrRNA), directs the SpCas9 nuclease to generate blunt double-strand breaks (DSBs) at the genomic DNA target site three bases upstream of Protospacer Adjacent Motif (PAM) sequence of ‘NGG’ (Jinek et al., 2012). The DSBs are repaired either by error-prone non-homologous end joining (NHEJ) resulting in insertion-deletion mutations (InDels) leading to gene knock-out or by precise, albeit inefficient, homology-directed repair (HDR) through which DNA insertions are achieved by providing an external donor repair template (DRT) (Atkins and Voytas, 2020; Dong and Ronald, 2021). In addition to Cas9, multiple other Cas variants with alternative PAM requirements have been identified and successfully utilized in plants expanding the range of DNA recognition (Kaya et al., 2016; Jia et al., 2017; Steinert et al., 2017; Zhang Y. et al., 2019; Veillet et al., 2020). Furthermore, base editors (BEs), including cytosine, adenine, and glycosylase BEs can precisely convert one target DNA base to another without a DSB. BEs rely on base excision repair, facilitating both transition and transversion mutations, and are increasingly being used in plant systems (Shimatani et al., 2017; Zong et al., 2017; Shan and Voytas, 2018; Zhang R. et al., 2019; Li et al., 2020; Zhao et al., 2020). In addition, prime editing (PE), a versatile “search-and-replace” strategy, was also developed (Anzalone et al., 2019) and optimized in plants (Butt et al., 2020; Lin et al., 2020; Tang et al., 2020; Xu et al., 2020). PEs copy desired edits incorporated into the PE gRNA (PegRNA) directly into the genomic DNA by target primed reverse transcription. With this existing toolbox of various CRISPR reagents, the biggest challenge in plant genome-editing remains to be the efficient delivery of these reagents into plant cells.

Several plant species have larger and more complex genome structures compared to other living systems. Polyploidy and genomic rearrangements are common in plants, and rigid cell walls surrounding the plant cells deter the entry of any foreign biomolecules. Furthermore, genetic transformation to deliver transgenes has only been established in a limited number of plant species and genotypes within each species. This is currently considered the biggest bottleneck in plant genome engineering. Gene-editing reagents are delivered into plants, most commonly as plasmid DNA constructs and predominantly by *Agrobacterium*-mediated transformation or particle bombardment are summarized in tables recently (Sandhya et al., 2020; Ghogare et al., 2021). In both methods, the plasmid DNA with CRISPR/Cas expression cassette is likely to get integrated into a random genomic site(s), leading to continued expression in host genomes. With the revision of the regulatory landscape of gene-edited lines in the US (USDA press release^1^) and across the world (Nadakuduti et al., 2018; Lassoued et al., 2021), developing gene-edited lines without integrating foreign genomic DNA into the host plant is gaining prominence. DNA-free delivery of *in vitro* transcripts (IVTs), pre-assembled ribonucleoprotein complexes (RNPs), or transient expression of plasmid DNA constructs delivered into protoplasts, and subsequent regeneration of gene-edited plants have been successful in several plant species (Liang et al., 2017; Andersson et al., 2018; González et al., 2020, 2021; Sidorov et al., 2021; Zhang et al., 2021). This review will focus on various advances in CRISPR delivery mechanisms in plants categorized into *Agrobacterium*-mediated delivery and breakthroughs for efficient and heritable mutagenesis and gene targeting (GT) in plants; particle bombardment mediated delivery of DNA, RNA, and protein biomolecules for plant gene-editing, and protoplast transfection and regeneration of transgene-free gene-edited plants. The ultimate goal in plant gene-editing is to establish highly efficient and species non-specific reagent delivery mechanisms for editing multiple targets simultaneously and achieve DNA-free gene-edited plants at scale.

### Breakthroughs in *Agrobacterium*-Mediated Delivery of CRISPR Reagents for Efficient and Heritable Mutagenesis and Gene Targeting

**FIGURE 1:**
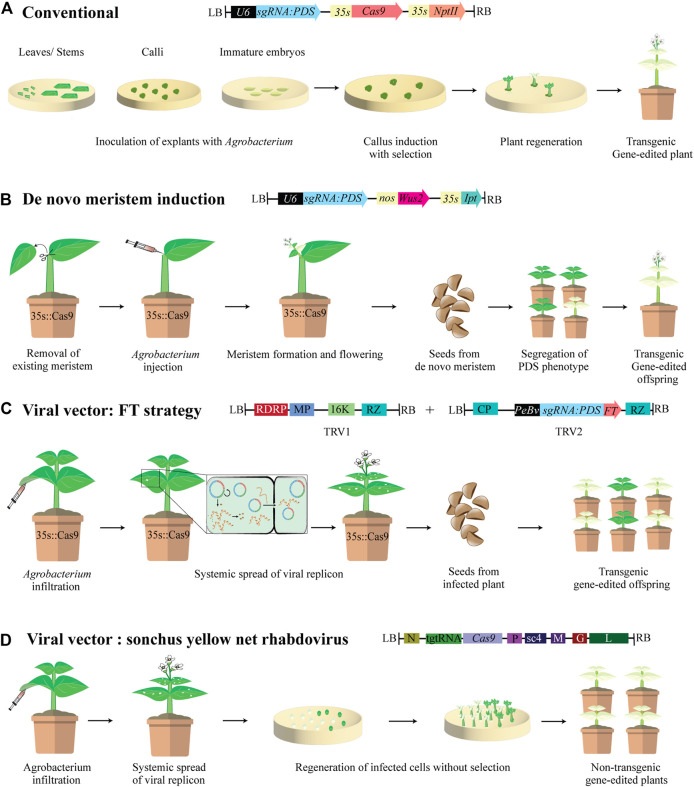
Agrobacterium mediated delivery of CRISPR gene-editing reagents in plants. **(A)** Conventional *Agrobacterium*-mediated transformation consisting of T-DNA carrying expression cassette for *Streptococcus pyogenes* Cas9 and kanamycin resistance gene *NptII,* both driven by cauliflower mosaic virus 35S promoter (CaMV 35S), and a single guide RNA (sgRNA) driven by the U6 promoter targeting the *phytoene desaturase* (*PDS*) gene. Explants are infected and co-cultivated with agrobacterium cultures, then placed on selective media for callus induction and regeneration. The resulting gene-edited lines are transgenic and have photobleaching phenotype. **(B)** *A. tumefaciens* T-DNA harboring sgRNA targeting *PDS* along with plant developmental regulators (DRs) *Wuschel2* (*Wus2*) driven by nopaline synthase (*nos*) promoter, and *isopentenyl synthase (ipt)* driven by 35S promoter are injected in Cas9 expressing soil grown plants after meristem removal. DRs induce new meristems at the wounded site and *pds* phenotype is visible in edited meristems. Offspring from seeds produced on *de novo* meristems show segregation for photobleaching phenotype. Maher et al. (2020) found that *de novo* meristems with bi-allelic mutations did not set viable seeds, and edited offspring are only recovered from meristems exhibiting mosaicism. **(C)** Tobacco rattle virus (TRV) is a bipartite RNA virus: TRV1 encodes replicases RNA-dependent RNA polymerase (RDRP), a movement protein (MP), a 16 KDa cysteine rich protein, and a ribozyme (RZ) and can independently replicate itself and move within the plant during infection. TRV2, encodes a coat protein (CP) and, a sgRNA targeting *PDS* fused to *Flowering locus T* (*FT*) driven by a pea early browning virus promoter (PeBv). FT is a mobile RNA which increases infection spread by reaching the shoot apical meristem (SAM). TRV1 and TRV2 are introduced into T-DNA regions of agrobacterium and infiltrated into 35S:Cas9 transgenic plants. Systemic infection of the plant leads to editing of somatic and germline cells thereby increasing heritability. Infected plants exhibit photobleaching and *pds* phenotype segregates in progeny. **(D)** Sonchus yellow net rhabdovirus (SYNV) is a negative-strand RNA virus encoding the core structural proteins nucleoprotein (N), phosphoprotein (P), and the large RNA polymerase (L), and Sc4 protein, matrix protein (M), glycoprotein (G) which are involved in cell-to-cell movement. The viral cassette is manipulated to express a Cas9 nuclease and a tRNA-gRNA-tRNA (tgtRNA) which is processed to release the sgRNA targeting the *PDS* gene by tRNA processing enzymes. Soil grown plants are infiltrated with agrobacterium harboring the SYNV plasmid. Explants from systemically infected leaves are prepared and placed on non-selective regeneration medium. Regenerants are then transferred to soil. Since Cas9 is delivered virally and SYNV does not integrate into the host genome nor have a DNA-phase, the resulting plants are non-transgenic.

*Agrobacterium*-mediated genetic transformation remains the principal means of delivering gene-editing reagents including CRISPR/Cas variants, base editing and prime editing reagents, into plants (Lin et al., 2020). This method typically involves inoculating the explants with *Agrobacterium* expressing gene-editing cassettes integrated into its T-DNA (Figure 1A). Upon infection of plant cells, the T-DNA containing the CRISPR cassette likely gets integrated into the host plant genome leading to stable genetic transformation. Transgene-free gene-editing has been achieved by transient expression of CRISPR reagents by regenerating events without employing selection (Chen et al., 2018). This is important for generating edited plants with no foreign DNA to avoid regulatory oversight and for vegetatively propagated plants, where segregating out the integrated transgene by making crosses is not feasible. *Agrobacterium* has a limited host range, and several plant species are recalcitrant to *Agrobacterium*-mediated transformation. Furthermore, the regeneration process involving tissue culture leads to undesirable somaclonal variations in edited lines. Floral dip method of transformation, only amenable to *Arabidopsis thaliana* and some related species (Clough and Bent, 1998; Lu and Kang, 2008) can generate transformed seeds, bypassing the need for regeneration. Other means of avoiding regeneration process include, the use of *A. rhizogenes*, which can drastically reduce time between reagent delivery and mutation evaluation, as well as widening the range of species transformed (Yoshida et al., 2015; Triozzi et al., 2021).

### Co-delivery of Developmental Regulators with CRISPR Reagents via *Agrobacterium* to Expedite and Improve Gene-editing Efficiency in Plants

Developmental regulators (DRs) are genes involved in dictating meristem identity in plants. Ectopic expression of DRs in plants has resulted in somatic embryogenesis, formation of embryos from somatic tissues (Lowe et al., 2016). Overexpression of DRs such as *Baby Boom* (*Bbm*) and *Wuschel2* (*Wus2*) enhanced regeneration and transformation frequency in both dicot and monocot plants (Srinivasan et al., 2007; Deng et al., 2009; Lowe et al., 2016). This phenomenon was leveraged to induce *de novo* meristems in somatic tissues by injecting *Agrobacterium* cultures co-delivering DRs and gene-editing cassettes directly into soil-grown plants (Figure 1B). *Wus2* and *Isopentenyl transferase* (*Ipt*), when co-delivered with gene-editing reagents by *Agrobacterium* injections into dicot plants generated meristems in somatic tissues with edits, enabling tissue culture free gene-editing (Maher et al., 2020). This can potentially be a high throughput and less tedious approach when Cas9 expressing plants are generated. Alternatively, Growth Regulating Factor (GRF) and GRF-interacting Factor (GIF) cofactor when expressed as GRF4-GIF chimera increased the speed and efficiency of regeneration (Debernardi et al., 2020). Co-delivery of GRF4-GIF chimera and CRISPR-Cas9 on the same T-DNA increased the regeneration efficiency in both monocots and dicots and produced fertile edited plants (Debernardi et al., 2020). The expression of DRs is extremely beneficial in plant species that are recalcitrant to regeneration or ones with long regeneration periods to reduce the time and cost of plant gene-editing.

### Viral Vectors and Mobile RNAs for Systemic Delivery of CRISPR Reagents for Heritable Gene-Editing

Recently, viral vectors showed promise for efficient delivery of CRISPR reagents into germline cells to achieve heritable and DNA-free gene-editing (Ali Z. et al., 2015; Ellison et al., 2020; Ma et al., 2020; Kujur et al., 2021; Li et al., 2021). Traditionally, heritable modifications are accomplished by stable expression of the CRISPR cassettes and generating transgenic lines through regeneration. Autonomously replicating viral vectors delivered into plants via *Agrobacterium* offer an alternative for heritable gene-editing in plants. RNA viruses don’t integrate into the plant genome but have lower cargo capacity impeding their use for Cas9 delivery. Tobacco rattle virus (TRV), a bipartite positive-strand RNA virus is widely used in plants. TRV mediated sgRNA delivery into Cas9 overexpressing lines by agroinfiltration has been optimized in dicots, albeit with low heritability of edits (Ali et al., 2015; Cody et al., 2017). To improve heritability, the endogenous mobile RNA *Flowering Locus T* (*FT*) has been fused to sgRNA to enhance mobility and facilitate systemic distribution within plant to reach germline cells (Figure 1C) (Ellison et al., 2020). Barley stripe mosaic virus (BMSV) has been engineered to deliver sgRNAs into wheat to achieve heritable genome editing. Furthermore, by co-infiltration of a pool of BMSV vectors harboring different sgRNAs resulted in multiplexed mutagenesis in the progeny (Li et al., 2021). Sonchus yellow net rhabdovirus (SYNV), a negative-strand RNA virus with higher cargo capacity, has been engineered to carry both Cas9 and sgRNA for DNA-free in planta editing (Figure 1D) (Ma et al., 2020).

### Enhancing Gene Targeting by *Agrobacterium*-Mediated Delivery of CRISPR Reagents

GT includes precise DNA modifications based on HDR using a DRT with homology to the host target DNA on both ends. DSBs generated by CRISPR/Cas reagents initiate the cell repair process. However, NHEJ is the predominant repair mechanism in plants cells to repair these DSBs as HDR is not active throughout the cell cycle. This, in combination with inefficient delivery of DRT to facilitate HDR, make GT very inefficient in plants. To increase GT frequencies, viral replicons including Bean Yellow Dwarf Virus (BeYDV) (Baltes et al., 2014; Butler et al., 2015; Čermák et al., 2015; Cermak et al., 2017; Wang et al., 2017; Vu et al., 2020) or wheat Dwarf virus (WDV) (Gil-Humanes et al., 2017) have been successfully used in several dicot and monocot plants. These viral replicons carrying the CRISPR expression cassette and DRT undergo rolling-circle replication in the host cells thereby increasing the abundance of nuclease and availability of DRT for HDR (Baltes et al., 2014). The GT event is not heritable if it doesn’t occur in the germline cells. To increase the heritability of GT, germline-specific promoters including the egg-cell, early embryo-specific promoter and pollen-specific promoters or promoters active in the shoot apical meristems (SAM) have been employed to drive Cas9 expression (Wang et al., 2015; Yan et al., 2015; Mao et al., 2016). Furthermore, to improve the efficiency of heritable in-frame gene insertions and amino acid substitutions by HDR, plants expressing Cas9 from germline-specific promoters are used for sequential transformation with HDR constructs containing DRT and sgRNA targeting the gene of interest. This led to an increase in GT efficiency of up to 9% (Miki et al., 2018). Since GT is a rare phenomenon, even with all the advances to improve efficiency, selection must still be employed to detect positive GT events. A *piggyBac* transposition system from T-DNA has been used to eliminate the GT selection marker from host plant genome. In this method, a transposon integrates into the host genome at TTAA element and excises without a footprint (Nishizawa-Yokoi et al., 2015). Recently, a novel marker elimination system was developed wherein the excision is based on I-SceI recognition site. By overlapping this recognition site on 5′ and 3′ homology arms of the DRT, seamless marker elimination and precise GT have been achieved (Endo et al., 2021). To this end, the same research group also developed a *piggyBac-*mediated transgenesis system to temporarily express CRISPR and selection marker cassettes from T-DNA with subsequent excision of *piggyBac* via transposase after successful editing and selection had occurred (Nishizawa-Yokoi and Toki, 2021).

### Biolistics for Delivery of CRISPR Reagents Into Plants as DNA, RNA, or Proteins

**FIGURE 2:**
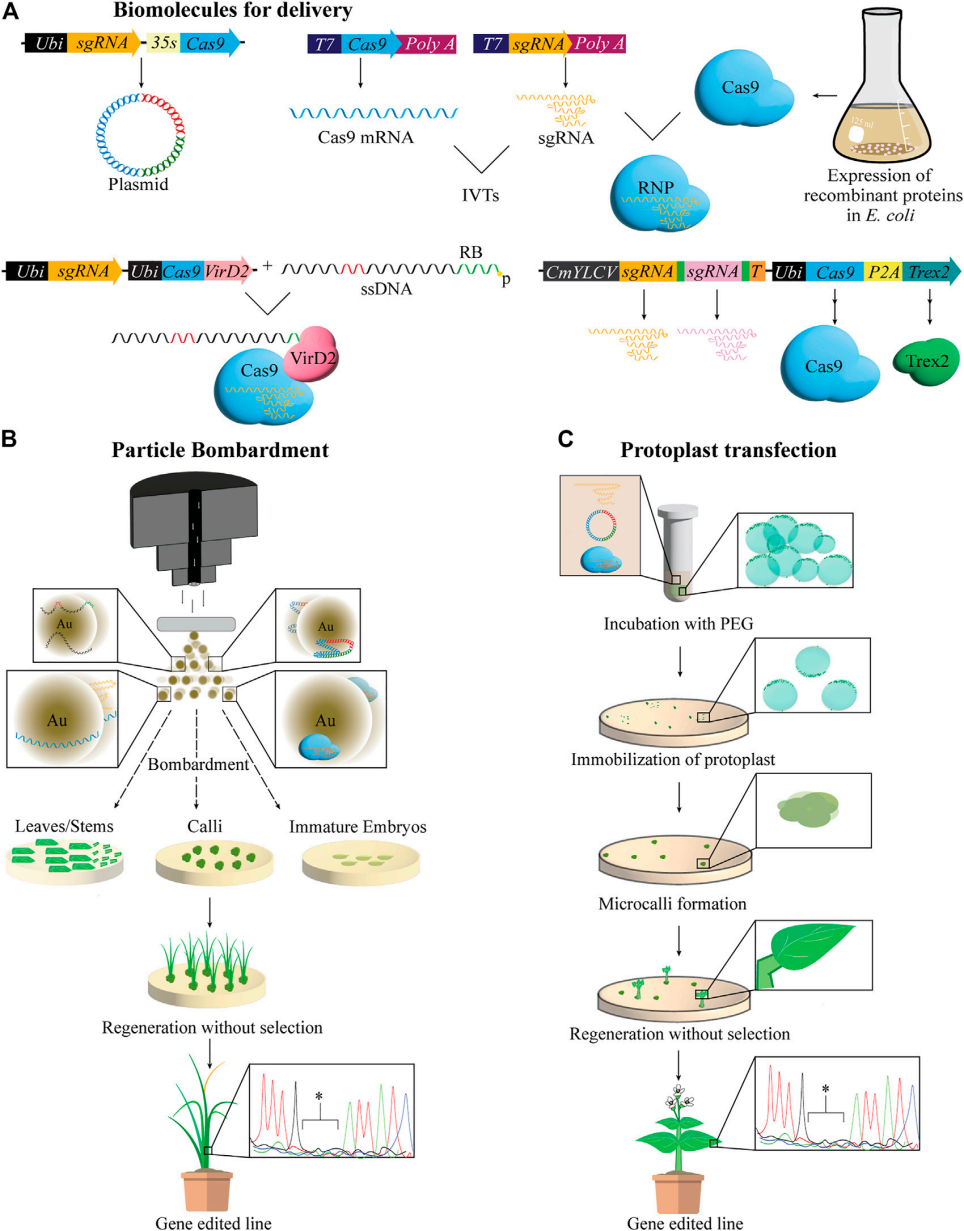
Biomolecules delivered via biolistics and protoplast transfections for regenerating gene-edited plants. **(A)** Biomolecules used for gene-editing are delivered into plants cells in a variety of forms including plasmid DNA, ssDNA, mRNA or ssRNA, prepared via *in vitro* transcription (IVT), and preassembled ribonucleic proteins (RNPs) using IVTs and recombinant proteins. Targeted mutagenesis and gene targeting (GT) can be enhanced by various mechanisms. For example, fusion of Cas9 to VirD2, one component of the agrobacterium relaxosome complex integral to the cleavage of T-DNA from the Ti plasmid, as well as its localization and integration in the plant genome, has been shown to increase homology-directed repair (HDR) mediated GT using a donor repair template (DRT). DRT in this case is a single stranded DNA (ssDNA) harboring the desired edits (red) and the canonical 25 bp right border (RB) sequence (green), and is delivered to the plant cell along with the Cas-VirD2 fusion protein. VirD2 will covalently bind the template, thus bringing it in close proximity to the DSB induced by Cas9. Delivering Trex2 exonuclease has also been shown to increase HDR as well as the efficiency of multiplex editing when sgRNA are co-delivered and processed by t-RNA system, illustrated by green boxes between sgRNA. p = phosphorylation. **(B)** Particle bombardment or biolistics, rely on the physical disruption of plant cell walls by metal particles, often gold, coated with ssDNA or dsDNA, IVTs or RNPs, which are introduced to the cell. Bombarded explants can be regenerated in tissue culture with or without selection to recover gene edited plants. Au = gold particles. **(C)** Protoplast transfection and regeneration is shown. polyethylene glycol (PEG) mediated transfection is the most common way to deliver biomolecules for gene-editing to protoplasts. Post transfection, protoplasts are immobilized on culture media where protoplasts undergo cell divisions to form microcalli, followed by shoot and root formation and finally resulting in regeneration of entire gene-edited plants. Editing at the target site is confirmed by sequencing represented in the chromatogram * = deletions.

Biolistics or particle-bombardment, is a common alternative for transforming plants recalcitrant to *Agrobacterium*-infection. It relies on physically breaching the plant cell wall and membrane with gold or tungsten microprojectiles coated with biomolecules accelerated to very high velocities. Biolistics offers the possibility of delivering a variety of cargo including plasmid DNA, ssDNA, RNA, or ribonucleic proteins (RNPs) assembled from IVTs and recombinant proteins (Figure 2A). Major drawbacks of biolistic delivery include random integration of cargo at multiple genomic sites when delivered as DNA and labor-intensive preparation of explants such as calli or immature embryos with the capability to regenerate.

### Advances in Biolistic Delivery for DNA-free Gene-Editing and Chromosome Engineering

Instead of plasmid DNA, bombarding RNPs was successfully demonstrated to produce transgene-free gene-edited lines in cereal crops (Svitashev et al., 2016; Liang et al., 2017; Banakar et al., 2019, 2020; Zhang et al., 2021) (Figure 2B). In addition, when a single base pair mismatch was present in the protospacer of sgRNA targeting homeologs, a dramatic decrease in off-target editing was observed with RNPs as compared to plasmid DNA delivery indicating high specificity of RNPs (Liang et al., 2017). Furthermore, RNPs also facilitated large heritable inversion of 75.5 Mb in maize chromosome 2, when assembled with guide RNAs flanking the junctions of the desired inversion (Schwartz et al., 2020). Such precise chromosomal engineering in invaluable to crop breeding. To avoid labor-intensive preparation of explants, *in planta* biolistic delivery using SAM as a target tissue (Hamada et al., 2017) for germline transmission was employed as an alternative (Hamada et al., 2018; Imai et al., 2020). Embryonic SAM exposed mature wheat seeds were bombarded with plasmid DNA expressing CRISPR cassettes to generate gene-edited lines (Hamada et al., 2018; Imai et al., 2020). Alternatively, when pollen was used as a target tissue to bombard plasmid DNA for gene-editing in *Nicotiana benthamiana*, the bombarded pollen retained fertility and delivered the cargo into the ovules (Nagahara et al., 2021). Furthermore, technical improvements have also been made to overcome variability between bombardments. A double-barreled gene gun in combination with cell counting software was developed to scale bombardment experiments with an internal standard, thereby reducing standard deviation between bombardments by half (Miller et al., 2021).

### Gene Insertion or Replacement by Intron Targeting and Determining Genomic Safe Harbors

To leverage the relatively more efficient NHEJ compared to HDR for targeted insertions, DNA fragments were inserted in selected introns such that any mutations by NHEJ would not affect protein-coding sequences of either endogenous or inserted genes. By bombarding calli with plasmids expressing pairs of sgRNA targeting adjacent introns of target genes and DRT harboring desired mutations flanked by the same intronic sgRNA sites, replacement of endogenous gene has been achieved at 2% frequency. Additionally, the gene replacement events were heritable (Li et al., 2016). Enhancers and promoters up to 2 Kb were introduced into the target site using these modified DRTs (Lu et al., 2020). Another strategy for targeted insertion by NHEJ is to determine the genomic safe harbors (GSH) in the host plant genome, within which integrations of transgenes do not cause any genic disruptions or adverse morphological effects. A 5.2 Kb carotenoid biosynthesis cassette was inserted at targeted GSH to generate marker-free rice with high carotenoid containing seeds and no-off target mutations observed (Dong et al., 2020).

### Enhancing HDR by Delivery of Transcript-Donor Templates or by VirD2 Relaxase-Cas9 Fusion

Recent advances in HDR by particle bombardment include delivery of ssDNA, including a canonical 25 bp right border (RB) sequence of T-DNA, as DRT co-delivered with a plasmid expressing Cas9-VirD2 fusion protein (Ali et al., 2020) (Figure 2A). Achieving a 20.8% HDR efficiency, this method relies on the ability of the VirD2 protein, an *Agrobacterium* virulence factor, to covalently bind the RB of DRT, thus bringing it in close proximity to the DSB induced by Cas9 (Figure 2A) (Ali et al., 2020). Other attempts to improve HDR include the delivery of DRT as transcripts. RNA-DRT was shown to result in higher HDR efficiency than DNA-DRT when delivered to rice calli, possibly due to the high stability of RNA:DNA complexes, resulting in edited rice with two desired point mutations in the *ALS* gene conferring herbicide tolerance (Li et al., 2019). This transcript-templated HDR (TT-HDR), approach improves not only HDR efficiency but also creates a DNA-free path to HDR-mediated gene-editing, which may avoid regulatory hurdles.

### Protoplasts Provide a Versatile System for DNA-free Genome Editing in Plants

Protoplasts are plant cells devoid of cell walls, which offer a versatile platform for DNA-free GE and a good transient system to evaluate the activity of gene-editing reagents before moving into a more-labor intensive transformation pipeline (Nadakuduti et al., 2019; Lin et al., 2020). Polyethylene glycol (PEG)-mediated transfection and electro-transfection are two common methods to deliver plasmid DNA, IVTs, or RNPs into protoplasts for transient expression of CRISPR cassettes. Subsequently, edited plants can be regenerated from transfected protoplasts by tissue culture procedures (Figure 2C). Plasmid DNA may integrate into the host genome randomly as filler DNA during protoplast transfection (Gorbunova and Levy, 1997; Kim and Kim, 2016). However, IVTs or RNPs offer DNA-free gene-editing by immediately editing the target site, bypassing transcription and translational machinery respectively in the cell and rapidly degrade (Liang et al., 2017, 2018; Andersson et al., 2018; González et al., 2020, 2020, 2020; Lee et al., 2020; Sidorov et al., 2021; Zhang et al., 2021). However, plant regeneration from protoplast remains unestablished in many plant species. In addition, somaclonal variations and genome instability is reported in regenerated lines (Fossi et al., 2019). Once efficient protoplast isolation, transfection, and regeneration have been established in a plant species, it could be a high throughput platform by combining with flow cytometry and omic analyses for optimizing gene-editing. Furthermore, multiplexing, editing multiple genes at a time has been achieved using protoplasts (Klimek-Chodacka et al., 2021; Nicolia et al., 2021; Yu et al., 2021; Zhang et al., 2021). By co-delivering Three Prime Repair exonuclease 2 (TREX2) and CRISPR/Cas9 into protoplasts, targeted mutagenesis using a multiplexing strategy was further improved (Weiss et al., 2020) (Figure 2A).

### Nanocarrier-Mediated Delivery of CRISPR/Cas Reagents in Plants

Nanotechnology has evolved in the past decade in the field of plant genetic engineering. Nanomaterials including carbon nanotubes (CNTs), carbondots, mesosporous silicon nanoparticles (MSNs) etc have been used to deliver biomolecules such as DNA, RNA, RNPs and proteins etc., discussed in recent reviews (Kumari and Singh, 2021; Mujtaba et al., 2021). Nanoparticle-mediated delivery of DNA and proteins into both nuclear and chloroplast genomes has been achieved in plants (Demirer et al., 2019, 2020; Kwak et al., 2019). Furthermore, Cre protein was previously delivered via MSNs for maize GE via *loxP* site demonstrating the feasibility of gene-editing (Martin-Ortigosa et al., 2014). Gene-editing using RNPs delivered by nanoparticles has been achieved in human cells (Wang et al., 2016; Lee et al., 2017; Mout et al., 2017). However, it has yet to be achieved in plants mainly due to high delivery efficiencies required for GE.

### Future Aspects of Delivering Plant-Gene Editing Reagents

Relying on tissue culture-based plant genetic transformation methods and inefficient reagent delivery mechanisms are the major bottle necks to overcome before we realize the full potential of gene-editing in plants. Current advancements in delivery mechanisms, including *de novo* meristem induction or use of viral vectors to circumvent tissue culture, rely on *Agrobacterium* for delivery and have been demonstrated only in dicots and need to be expanded to monocots. Delivering repair templates for HDR through these innovative methods is also a future possibility. Furthermore, smaller sized Cas9 alternatives would overcome the cargo capacity of some of these viral vectors. *Agrobacterium,* however, has a narrow host range for infection and several species are recalcitrant to *Agrobacterium* transformation. Particle bombardment has been shown to be better equipped for co-delivery of cargo for simultaneous editing than *Agrobacterium* and is universally applicable to all plant species and cell types (Kuang et al., 2020). Chromosomal inversions achieved via bombardment could revolutionize breeding by unlocking regions for chromosomal cross overs, creating novel linkage groups and facilitating targeted recombination to maximize genetic gain in crops. However, complex segregation patterns of DNA integrated in bombarded plant genomes might complicate downstream uses of transformed plants. *Agrobacterium* and biolistic transformation of pollen also bypasses regeneration but often results in pollen with lower viability (Wang et al., 2008; Zhao et al., 2017). In addition, pollen-tube transformations may result in chimerism (Ali A. et al., 2015). While pollen magnetofection has improved on these drawbacks (Zhao et al., 2017), its application remains constrained to dicots (Vejlupkova et al., 2020). The prospects of nanoparticles as delivery engines for plant genome editing are also encouraging (Demirer et al., 2021) and further advances are essential to facilitate plant gene-editing.

## Footnotes

1
https://www.usda.gov/media/press-releases/2018/03/28/secretary-perdue-issues-usda-statement-plant-breeding-innovation.
